# Dynamic Responses of Human Skin and Fascia to an Innovative Stimulation Device—Shear Wave Stimulation

**DOI:** 10.3390/biomimetics9080475

**Published:** 2024-08-06

**Authors:** Na Qiao, Lucas Ouillon, Alexandre Bergheau, Virginie Dumas, Coralie Privet-Thieulin, Jean-Luc Perrot, Hassan Zahouani

**Affiliations:** 1 Univ Lyon, Ecole Centrale de Lyon, CNRS, ENTPE, LTDS, UMR5513, 69130 Ecully, Francehassan.zahouani@ec-lyon.fr (H.Z.); 2 Univ Lyon, Ecole Centrale de Lyon, CNRS, ENTPE, LTDS, UMR5513, ENISE, 42023 Saint-Etienne, France; 3 ECE, OMNES Education Research Center, 75015 Paris, France; 4 Département de Dermatologie, Centre Hospitalier Universitaire de Saint-Etienne, 42055 Saint-Etienne, France

**Keywords:** shear wave stimulation, human skin, fascia, mechanotransduction, mechanical behaviors, massage

## Abstract

Exposure to mechanical stimuli such as pressure and stretching prompts the skin to undergo physiological adaptations to accommodate and distribute applied forces, a process known as mechanotransduction. Mechanotherapy, which leverages mechanotransduction, shows significant promise across various medical disciplines. Traditional methods, such as massage and compression therapy, effectively promote skin healing by utilizing this mechanism, although they require direct skin contact. This study introduces a novel contactless modality, Shear Wave Stimulation (SWS), and evaluates its efficacy compared to traditional massage in eliciting responses from human skin and fascia. Fifteen healthy volunteers received SWS, while another fifteen volunteers received massage. Tests of skin mechanical properties revealed significant enhancements in skin shear modulus for both methods, showing an increase of approximately 20%. Additionally, deformation analysis of ultrasound images showed distinct responses of the skin and fascia to the two stimuli. SWS induced extension in the dermis (∼18%), hypodermis (∼16%), and fascia (∼22%) along the X and Y axes. In contrast, massage compressed the skin layers, reducing the dermis by around 15% and the hypodermis by about 8%, while simultaneously stretching the superficial fascia by approximately 8%. The observed extension across the entire skin with SWS highlights its potential as a groundbreaking contactless approach for promoting skin healing. Furthermore, the differing responses in blood flow reaffirm the distinct stimulation modes of SWS and massage. These findings establish a foundation for future innovative skin therapy modalities.

## 1. Introduction

Human skin, as known, is a stratified tissue composed of the epidermis, dermis, and hypodermis [1], with thickness varying across different body parts. The epidermis is constructed from various differentiation stages of keratinocytes stacked together [2]. The dermis is predominantly composed of connective tissue, where collagen fibers form a cross-linked network responsible for the skin’s tensile properties, and elastic fibers create a three-dimensional (3D) network imparting elasticity to the skin [3,4]. Fibroblasts secrete the extracellular matrix (ECM) components of connective tissues, and daily mechanical forces such as walking, exercise, and massage influence the synthesis and degradation of collagen and elastin [5]. The hypodermis generally comprises loose connective tissue anchoring the skin to the deep fascia and adipose tissue to store the fat [6]. Superficial fascia can be found in this layer [7].

With aging, the metabolism of the skin slows down, and the production of collagen fibers becomes slow. The composition of the ECM deteriorates, and water is lost. Consequently, this leads to changes in skin structure and subsequently affects the mechanical properties of the skin [8,9]. The ability of the skin to respond to changes in mechanical load may be reduced [10]. The overall resilience of the skin decreases, meaning it is more likely to sustain damage from mechanical loads and less likely to heal quickly. The rate at which skin recovers from mechanical deformation slows down, leading to prolonged periods of distortion or damage after stress is applied.

Beneath the skin lies another crucial type of connective tissue, the fascia, whose role is increasingly appreciated [11]. The fascial system connects the entire body, allowing it to function systematically [12,13], thereby manipulating, resisting, and distributing mechanical forces throughout the body.

The skin separates the human body from the external environment and constantly adapts and senses a wide range of mechanical cues in the entire life [14]. When the skin is exposed to external stimuli, the cells within it undergo a process known as mechanotransduction, where mechanical forces are converted into biochemical and electrochemical signals. This mechanism plays a crucial role in regulating cellular functions such as proliferation and metabolism, ultimately leading to changes in tissue properties [15,16,17]. Massage and beauty devices leverage mechanotransduction to relieve pain, assist in injury recovery, improve mechanical behaviors, and delay the aging of the skin [18]. Similarly, mechanical stimulation can likewise trigger mechanotransduction in the fascia, thus inducing a more youthful collagen structure [19,20,21]. However, to date, these techniques require direct contact with the skin, making them unsuitable for treating conditions such as burned skin, pressure ulcers, and other diseases that damage the skin’s surface. Additionally, few studies have reported the quantitative impact of mechanical stimulation on the mechanical properties of skin and fascia in vivo.

A non-invasive method for stimulating the skin and fascia using shear waves and a small portion of compression waves generated by air has been developed. In previous studies, we utilized this modality, shear wave stimulation (SWS), to stimulate reconstructed skin and observed enhancements in its mechanical properties. These improvements included an increased shear modulus of skin substitutes, an increase in the number and diameter of fibroblasts, and a higher density of collagen and elastin [22].

Simultaneously, the use of imaging technology, such as ultrasonography, has become increasingly important in tissue examinations. Ultrasonography allows for the visualization and quantification of connective tissue and muscle structures, as well as the dynamic responses to local mechanical perturbations [23,24,25]. By employing ultrasonography, it is possible to analyze the stimulation principles of SWS and traditional stimulation instruments, thereby gaining a better understanding of their intended application. Besides, Utilizing Laser Doppler to measure local subcutaneous blood flow perfusion enables an understanding of the therapeutic mechanisms underlying the physiological effects of mechanical stimulation on microcirculation in vivo [26,27].

Therefore, in comparison to a commercial massage device, SWS was applied to the right inner forearm of 15 volunteers over a period of two weeks to investigate the responses of human skin and fascia. We demonstrated that the shear modulus of the skin increased after both stimuli. However, unlike the existing massage method, SWS simultaneously stretched multiple skin layers (dermis, hypodermis) and the fascia, while having minimal impact on skin blood perfusion. This unique stimulation pattern positions SWS as a potential treatment modality for wound healing. Additionally, age did not significantly affect the beneficial effects of SWS on the skin, highlighting its potential applicability across different age groups.

## 2. Materials and Methods

### 2.1. Participants

**Figure 1:**
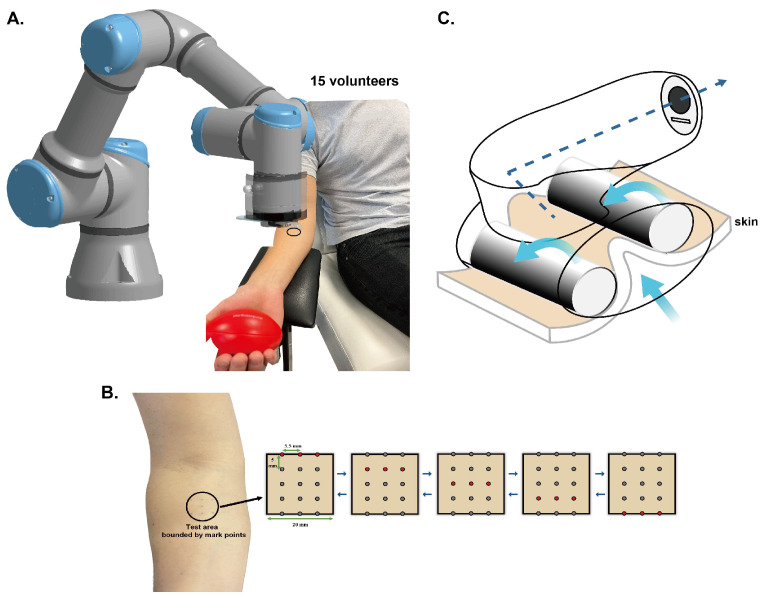
Illustration of participant posture (**A**), Y-movement of SWS (**B**), and the principle of the massage device (**C**).

The study was performed on the right volar forearms of participants. We recruited four groups of volunteers: 8 youths (5 male and 3 female) aged 20–35 (Group A) and 7 elders (4 male and 3 female) aged 50–65 (Group B) with both genders for SWS; 8 youths (6 male and 2 female) aged 20–35 (Group C)and 7 elders (3 male and 4 female) aged 50–65 (Group D) for a traditional massage device (LPG Wellbox^®^, Paris, France). All the volunteers are in good health conditions and are non-smokers. Their phototype is from I to III, and the experimental skin area is without scars and tattoos. To ensure that the subjects have similar behavior in daily life during the study. For example, they usually worked in the office and did not exercise much daily. The volunteers had a Body Mass Index (BMI) between 18 and 25 kg/m^2^ and were nonsmokers. The volunteers did not apply cosmetic products to their test area and avoided shaving. Before the test, all the volunteers did not consume irritating food and drinks and needed to sit undisturbedly in the experimental room with a constant temperature and humidity for at least 10 min as an acclimation period [28]. Afterward, the participants were placed in a dental chair, with their backs firmly against the slightly sloping backrest, their legs stretched out naturally and uncrossed on the chair seat. A height-adjustable armrest was placed next to the chair so that the volunteers could put their right anterior forearms on it, while an oval ball was lightly grasped in their hand to maintain a relaxed and steady position (Figure 1A). They needed to maintain this position throughout the experiment to avoid systematic errors. Informed consent was provided to all subjects. They were utterly informed of experimental procedures prior to the test and agreed to participate. The procedures of this study complied with the latest Declaration of Helsinki.

### 2.2. Devices and Protocols

#### 2.2.1. Mechanical Stimulation Devices

- Shear wave stimulation (SWS)

The stimulation method of SWS has been thoroughly described in the earlier study. By utilizing air, shear and compression waves are generated within the skin, leading to alterations in cellular responses, resulting in changes in the mechanical properties of the skin [22]. The stimulation parameters of SWS in vivo differ from that of SWS in vitro: the internal diameter of the outlets is bigger (1.19 mm); the interval distance of Y-movement is 5 mm; the opening time was increased to 75 ms; and each air pressure is 4 bar (Figure 1B).

- Traditional massage device (Wellbox)

To compare with SWS, the commercial massager, Wellbox, was used as another stimulation device that creates absorption by a bump to keep the skin fold and holds two rollers to allow the device to move over the skin on account of no rhythm mode and head ①. The power is set to 6 (Figure 1C).

#### 2.2.2. Characterizations Methods

- The shear modulus of the skin: UNDERSKIN

UNDERSKIN was described in detail in previous studies [22,29].

In this study, the impact pressure is set to 4 bar, and the time is 7 ms. The optical sensor is located at a distance of 0.3 mm from the solicitation point along the central Y-axis of the test area. The measurements were repeated five times in each test.

- The deformation of different layers of skin and fascia: Ultrasound and deformation analysis

Supersonic Mach 30 (Supersonicimagine, France) and an analytical approach to ultrasound images were engaged to calculate the extension rate of different skin layers and fascia. The B-mode of Supersonic Mach 30 was used to access the two-dimensional anatomy of skin and fascia with a 1.6 cm depth scale and transducer LH20-6. The transducer was positioned along the central Y-axis of the test area. Imaging parameters were set up as follows: (1) Optimization: General; (2) TissueTuner™: 1480 m/s; (3) Dynamic Range: 63 dB; (4) 2D map: 10; (5) Persistence: Low; (6) PRF: Low; (7) AutoTGC offset: 0 dB; (8) SuperRes™: 4; (9) Acoustic Power: 0 dB; (10) Zoom Percentage: 118%; (11) Sector: Large with 59 Hz frequency. The imaging parameters and test area were consistent in each measurement, and three images were acquired in each test.

The average wavelengths of the dermis, hypodermis, and superficial fascia in the X and Y directions were extracted from the ultrasound images [30]. The $\lambda_{0}x$ and $\lambda_{0}y$ denote the average wavelengths of each layer before the mechanical stimuli, while after stimulations are $\lambda_{1}x$ and $\lambda_{1}y$. The deformations are quantified at the scale of the microstructure of the tissue image. The methodology uses the quantification of the centroid frequencies of the image. The centroid spatial frequencies are determined in the frequency domain of the Fourier transform of the image. Centroid measurement consists of determining where the spectral center of gravity of the image is located. Its value corresponds to a frequency (in Hz), on either side of which the energy of the spectrum is equally distributed. If f(x,y) is the ultrasound image before or after mechanical stimulation, and if we note the Fourier transform of the image as

(1) $$TF(f\left(x,y\right):TF\left(fx,y\right)=\sum_{k=1}^{N}\sum_{t=1}^{M}\left[f\left(x,y\right)e^{-j2\pi (\frac{ux}{M}+\frac{vy}{N})}\right]$$

with $j^{2}=-1$, M and N the number of pixels of the image in the x and y directions.

We denote the spatial frequencies in the two x and y directions as

(2) $$u_{i}=\frac{i}{N△x},v_{j}=\frac{j}{M△y}$$

i and j varying from 1 to N and from 1 to M. △x and △y are the steps between two pixels in the x and y directions of the image.

The spatial frequency spectrum extends from low frequencies to

(3) $$i=1,j=1;u_{1}=\frac{1}{N△x},v_{1}=\frac{1}{M△y}.$$

And at high frequencies for

(4) $$i=\frac{N}{2},j=\frac{M}{2};u_{\left(N/2\right)}=\frac{1}{2△x},v_{\left(M/2\right)}=\frac{1}{2△y}$$

We quantify the effect of mechanical stimulation at the microstructure level of the tissue image, by determining the centroid frequencies between low and high frequencies as:

(5) $$u_{x}^{c}=\frac{(\sum_{i=1}^{N}\sum_{j=1}^{N}\left[u_{x}TF\left(f\left(x,y\right)\right]\right)}{\sum_{i=1}^{N}\sum_{j=1}^{N}\left[TF\left(f\left(x,y\right)\right]\right)},v_{y}^{c}=\frac{(\sum_{i=1}^{N}\sum_{j=1}^{N}\left[v_{y}TF\left(f\left(x,y\right)\right]\right)}{\sum_{i=1}^{N}\sum_{j=1}^{N}\left[TF\left(f\left(x,y\right)\right]\right)}$$

The centroid wavelengths of the image along the x and y axes are given as:

(6) $$\lambda_{x}^{c}=\frac{2\pi }{u_{x}^{c}},\lambda_{y}^{c}=\frac{2\pi }{v_{y}^{c}}$$

If we define the wavelengths of the image before stimulation as $\lambda_{x0}^{c}$ and $\lambda_{y0}^{c}$, and the wavelengths of the image after stimulation as $\lambda_{x1}^{c}$ and $\lambda_{y1}^{c}$.

The deformation ratios in X and Y before and after stimulations are defined as:

(7) $$\epsilon_{x}=\frac{\lambda_{x1}^{c}-\lambda_{x0}^{c}}{\lambda_{x0}^{c}},\epsilon_{y}=\frac{\lambda_{y1}^{c}-\lambda_{y0}^{c}}{\lambda_{y0}^{c}}$$

The wavelengths before mechanical stimulation served as a reference for comparison with those after SWS and Wellbox massage to quantify the deformation ratios. A negative deformation ratio indicates compression of the skin and fascia, while a positive deformation ratio indicates extension. This statistical analysis of skin and fascia provides insights into the fundamental mechanisms of mechanical stimulation.

- Blood flow: PeriFlux System 5000

Microenvironmental blood flow of the massaged skin area was measured by PeriFlux System 5000 (PERIMED, Järfälla, Swedish). In virtue of the Laser Doppler, PerFlux System 5000 (Laser Doppler Perfusion Monitor (LDPM) unit) transmits laser light through an optical fiber, and some of the light is absorbed after the laser beam is scattered by the target tissue. The wavelength of the laser that hits blood cells has changed. The intensity and frequency distribution of these wavelength changes are directly related to the number and moving speed of blood cells in the monitoring volume. Through the receiving fiber, this information is recorded and converted into electrical signals for analysis. As a result, the microenvironmental blood flow in the assay area was monitored.

The non-invasive Laser Doppler perfusion monitor probe 407 was used. The time constant is 0.2, and the experimental parameters are “auto connected”. Measurements were taken at the 3 points along the central Y-axis of the test area.

#### 2.2.3. Protocols

- Protocol for SWS

PeriFlux System 5000, UNDERSKIN, and Supersonic Mach 30 were used in sequence for characterizations before the SWS, followed by 10-min SWS. After waiting for 1 min, the PeriFlux System was employed again. We repeated the daily protocol for two weeks.

- Protocol for Wellbox

Same as SWS, the three characterizations were applied before the massage in the morning. A 2-min massage with Wellbox was pursued. The PeriFlux System was operated after 1 min. In the afternoon, another 2-min massage was performed on the same skin area of the participants, before and after which only the PeriFlux System was employed. It is worth noting that the interval between these two massages should be at least 4 h. This quotidian protocol was carried out lasting two weeks as well.

### 2.3. Statistical Analysis

The data from 15 volunteers are presented using box plots. The elasticity data of Subjects A and B are depicted in line and symbol graphs. Blood flow data of Subjects A and B are shown as mean ± standard error of the mean (SEM). Paired samples *t*-tests were used for statistical comparisons before and after stimulation, while independent-sample *t*-tests were employed to compare data between the young and old groups, as well as between SWS and Wellbox groups. Significance was considered for *p* < 0.05.

## 3. Results

### 3.1. Response in Skin’s Mechanical Behavior

**Figure 2:**
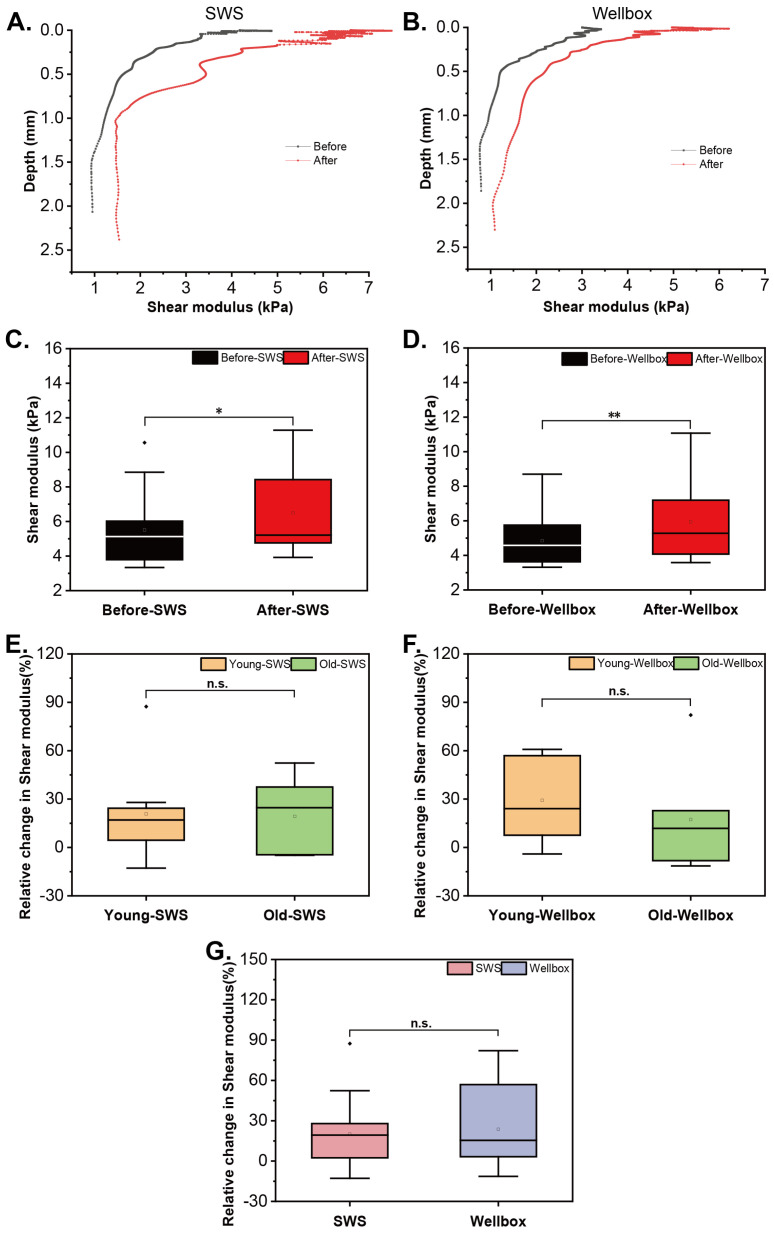
Shear modulus of the skin. Representative depth-shear modulus curves before and after SWS (**A**) and Wellbox (**B**). Comparison of shear modulus before and after SWS (**C**) and Wellbox (**D**) in 15 volunteers. Differences in the effects of SWS (**E**) and Wellbox (**F**) between young and aged groups in skin shear modulus. Comparison of the effect of the two stimulation methods on skin shear modulus (**G**). Significant differences between groups (*p* < 0.05) are denoted by an asterisk; (*p* < 0.01) are denoted by two asterisks; n.s.: not significant.

The shear modulus at different depths of the skin was measured using the UNDERSKIN technique. One of the volunteers, Subject A, was selected as an example to evaluate the impact of SWS on skin mechanical properties. The shear modulus was compared before and after two-week SWS, and the results showed a substantial increase in skin shear modulus, with a 52.4% improvement (Figure 2A). Further analysis of all the volunteers’ data using paired sample *t*-tests confirmed a significant augment in shear modulus after SWS (Figure 2C; n = 15; *p* = 0.02), with a 17.9% increase.

Similarly, to demonstrate the effect of Wellbox, the shear modulus obtained from UNDERSKIN on the first (before) and last (after) days of Subject B was employed (Figure 2B). The Wellbox led to an 82.1% increase in the shear modulus of Subject B’s skin. Generally, a considerable increase in the shear modulus of the 15 volunteers was observed (Figure 2D; *p* = 0.004), being consistent with literature reports [31,32]. The growth rate was 22.5%.

The relative changes in skin shear modulus resulting from SWS and Wellbox are depicted in Figure 2E,F. Aging skin did not exhibit significant impacts in the effects of SWS and Wellbox in terms of the mechanical behavior of the skin.

As depicted in Figure 2G, both SWS and Wellbox demonstrated similar influences on skin shear modulus. Both techniques resulted in the increased mechanical behavior of the skin, with no significant difference in their effects.

### 3.2. Response in Deformation of Skin and Fascia

**Figure 3:**
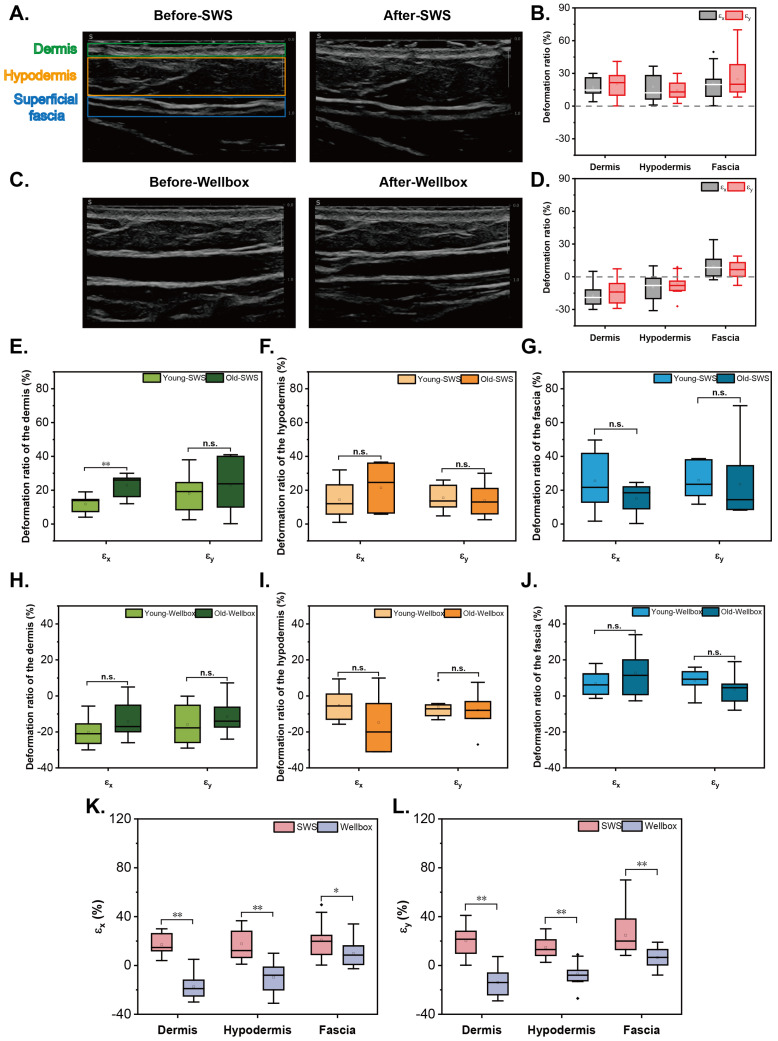
Deformation ratio of the skin and fascia. Representative Ultrasound images of Subject A (**A**) and Subject B (**C**). Deformation ratios before and after SWS (**B**) and Wellbox (**D**) in 15 volunteers. Differences in the effects of SWS (**E**–**G**) and Wellbox (**H**–**J**) between young and aged groups in deformation of dermis, hypodermis, and fascia. Comparison of the effect of the two stimulation methods on the deformation of skin and superficial fascia in X (**K**) and Y (**L**) directions. Significant differences between groups (*p* < 0.05) are denoted by an asterisk; (*p* < 0.01) are denoted by two asterisks; n.s.: not significant.

The ultrasound images before and after SWS of Subject A are presented in Figure 3A. Combining the deformation theory, the deformation ratios of the dermis, hypodermis, and fascia were analyzed. After two-week SWS, the layers of the dermis, hypodermis, and fascia in the 15 volunteers were extended in X and Y directions (Figure 3B). The mean values and SEM of deformation ratios are quantified in Table 1. The findings suggest that shear waves have the ability to stretch the dermis, hypodermis, and superficial fascia in both the X and Y directions, with similar degrees of extension observed in these three layers.

The ultrasound images of Subject B captured before and after Wellbox are illustrated in Figure 3C. By integrating the deformation theory, the deformation ratios of the 15 volunteers before and after the massage are outlined in Figure 3D. The dermis and hypodermis of these 15 participants experienced compression in both the X and Y directions, while an extension of the fascia was observed. The mean values and SEM of the deformation ratios are quantified in Table 2. These results indicate a shift in the state of the tissues due to the massage mechanism, progressing from compression to extension as we move from the upper to the lower layers.

In addition, the differences rendered by aging in deformation ratios of the skin and fascia were not significant generally, except for the extension of the dermis in the X-axis under SWS (n = 15; *p* = 0.003) (Figure 3E–J).

To compare the two stimulation modalities, SWS exerted a consistent effect on all layers of the skin and superficial fascia, leading to the extension of these tissue layers in both directions. On the other hand, Wellbox applied compression to the contacted tissue layers while stretching the deeper layer (Figure 3K,L). There is a significant difference in the stimulation mechanism between these two methods for the skin and fascia.

### 3.3. Respose in Blood Flow

**Figure 4:**
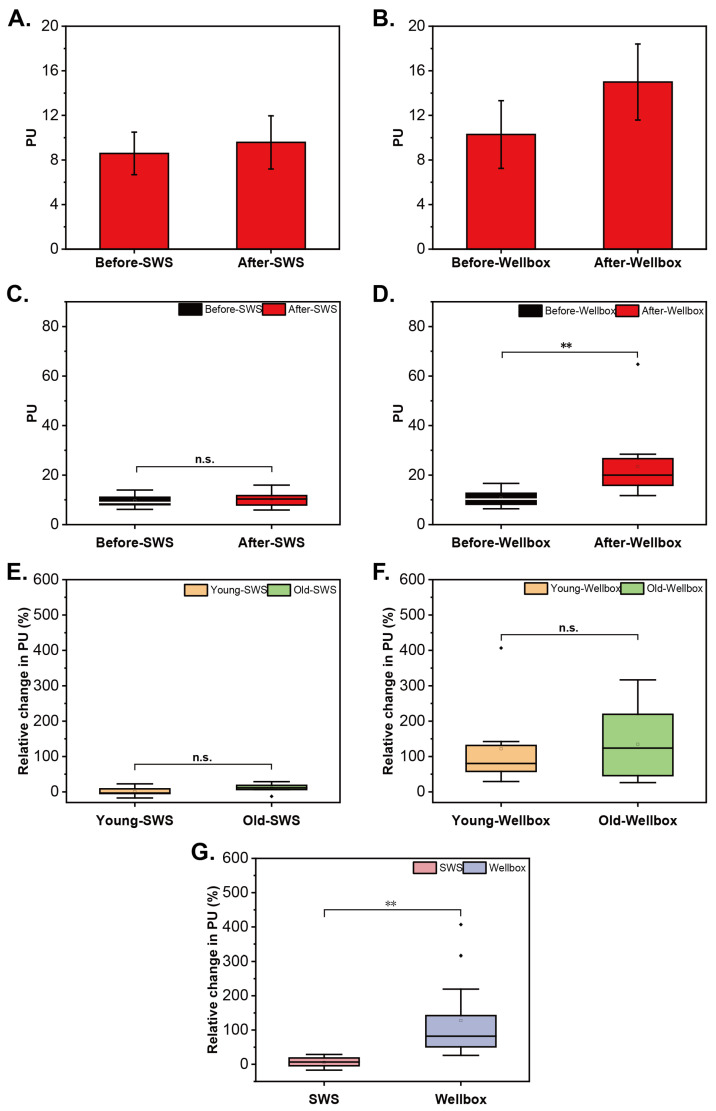
Blood flow of test area. Representative of the comparison of the mean blood flow measured before and after SWS (**A**) and Wellbox (**B**). Comparison of blood flow before and after SWS (**C**) and Wellbox (**D**) in 15 volunteers. Differences in the effects of SWS (**E**) and Wellbox (**F**) between young and aged groups in skin blood flow. Comparison of the effect of the two stimulation methods on skin blood flow (**G**). Significant differences between groups (*p* < 0.01) are denoted by two asterisks; n.s.: not significant.

The average PU value of the three points within the test zone was calculated to represent the result of each measurement. To investigate the modification of cutaneous blood flow before and after SWS, the mean value of all the measurements prior to SWS and all the measurements after SWS were employed. Figure 4A illustrates a slight increase in skin blood perfusion with a relative change of 11.5% in Subject A. However, the blood perfusion remained unaffected after SWS in 15 participants (Figure 4C; n = 15; *p* = 0.2).

As shown in Figure 4B, the skin perfusion boosted after the stimulation of Wellbox, showing a growth rate of 45.8% in Subject B. Summarizing the data of 15 volunteers, there was a marked difference in skin blood flow before and after the massage, and the difference is extremely large (Figure 4D, *p* = 0.001).

In comparison to the young skin, the aged skin did not yield any significant difference in the effect of SWS and Wellbox in blood perfusion (Figure 4E,F).

The effects of SWS and Wellbox on blood flow in the test skin area showed a significant difference, and the magnitude of this difference was substantial (Figure 4G; n = 15; *p* = 0.000; Cohen’s d = 1.573).

## 4. Discussion

Advancements in reconstructed skin research have highlighted the potential of shear wave stimulation (SWS) in enhancing skin mechanical properties [22]. This study amalgamated data from both younger and older participants to explore the versatile applications of SWS. The results indicate that SWS implementation correlates with heightened shear modulus in the human skin, consistent with in vitro findings and resembling the effects of commercial massage devices. SWS, thus, presents a promising method for dermatological treatments that require enhanced skin responsiveness to external forces, such as in the early phases of wound healing and skin aging.

On the other hand, aging induces notable changes in skin mechanical behaviors [33,34,35], underscoring the importance of investigating age-related phenomena. Consequently, comparative analyses between younger (Group A) and older participants (Group B), as well as between the corresponding groups for traditional massage (Groups C and D) were conducted. The findings revealed no significant variations in response to identical mechanical stimuli among the different age cohorts. This lack of distinction may be attributed to the limited sample size and substantial individual differences in skin condition, leading to insufficient data to discern disparate responses to the stimuli. Nonetheless, this result presents the versatility of SWS across different maturity levels of the skin.

Using the Extension model, we analyzed the deformation of skin and superficial fascial layers before and after SWS and massage from ultrasound images. The observed disparities between the results of SWS and massage can be attributed to the distinct principles underlying the two stimulation methods. SWS, a non-contact technique utilizing shear waves, does not involve skin compression. Conversely, the massage head applies compression to the skin surface, which, combined with the rolling action of the massager, stretches the superficial fascia beneath the skin. Notably, significant differences in the deformation of the dermis in the X direction were observed: post-SWS, older skin exhibited a higher propensity for stretching in the X direction.

Previous in vitro assessment of the effects of SWS on skin microstructure evidenced that SWS induced a significant increase in collagen and elastin density within skin substitutes, along with notable augments in fibroblast morphology and population [22]. Incorporating this study’s in vivo result regarding the analysis of stimulation mode of SWS positions that SWS carries the prospect as a therapeutic intervention for promoting wound healing processes and addressing age-related skin concerns.

Several studies have highlighted the efficacy of massage, particularly vigorous techniques, in enhancing blood perfusion, with mechanical massage proving most impactful [36]. Additionally, it’s noted that the rise in blood flow velocity is transient. The massage device used in our study operates on a principle similar to vacuum massage, creating localized suction through a pump mechanism. Literature suggests that vacuum massage can significantly augment cutaneous blood flow and induce vasodilation, making it applicable to scar therapy [32,37]. This study reveals that Wellbox, the mechanical massage device, did raise the blood flow temporarily under the skin, which is uniform with the impacts declared in the existing literature. However, certain massage techniques are theorized to augment skin blood flow by dilating capillaries in response to mechanical stimuli, leading to localized warmth. Nonetheless, this effect may be superficial, temporary, and of limited magnitude [38], unrelated to the primary therapeutic objectives of massage therapy.

Notably, SWS did not impact blood perfusion. One potential explanation is the use of cold air in SWS, as temperature significantly influences blood flow measurement. Furthermore, the blood flow results underscore the divergent stimulation patterns between SWS and Wellbox as well. The SWS uses air-generated shear waves within the skin to stimulate it non-contactly, whereas the massage device lifts the skin by suction and then uses rollers to move back and forth over the target area. As mentioned earlier, the observed increase in blood flow following massage may hold more relevance in scar treatment. Conversely, SWS appears more conducive to repairing damaged skin, particularly in stemming bleeding during the initial stages of wound healing.

Moreover, our findings indicate no significant disparity in skin blood flow response between youthful and aged participants.

In the end, it’s important to state that this study exclusively demonstrates, in line with in vitro findings, that the SWS has the ability to elevate the shear modulus of skin in vivo. Meanwhile, it showcases SWS’s distinct stimulation mode attributable to its contactless nature. Regarding its potential application in dermatological therapy, further research is warranted to explore its full scope. Additionally, it’s worth emphasizing that the stimulation parameters of SWS, such as air pressure and duration of application, can be tailored to suit various treatment scenarios.

## 5. Conclusions

In summary, at first, through investigating the dynamic responses of human skin and fascia to SWS, we demonstrated the versatile application of SWS, demonstrating its viability in vivo. It can enhance the mechanical behaviors of the skin, simultaneously stretching the skin and fascia without altering blood flow. This demonstrates its potential ability to promote skin healing. Furthermore, SWS allows for customizable stimulus frequency adjustments to cater to diverse needs. The distinct stimulation parameters employed between reconstructed skin and human skin highlight the necessity for precise adjustments in stimulus intensity to optimize skin mechanical properties. Moreover, the mobility of SWS, coupled with adaptable robotic arm dimensions, enables targeted stimulation of specific regions.

On the other hand, our examination of aging effects on the skin’s dynamic response revealed valuable insights. Despite the limited sample size, our findings suggest that older skin exhibits a greater propensity for extension in the X direction following mechanical stimulation. Meanwhile, it indicates the potential utility of SWS across different age groups.

## Figures and Tables

**Table 1:** Deformation ratios before and after SWS in 15 volunteers. Unit: %.

	Dermis	Hypodermis	Fasica
$\epsilon_{x}$	16.98 ± 2.06	17.63 ± 3.16	20.65 ± 3.76
$\epsilon_{y}$	20.37 ± 3.38	14.69 ± 2.16	24.78 ± 4.3

**Table 2:** Deformation ratios before and after Wellbox in 15 volunteers. Unit: %.

	Dermis	Hypodermis	Fasica
$\epsilon_{x}$	−17.33 ± 2.46	−9.61 ± 3.32	9.67 ± 2.56
$\epsilon_{y}$	−13.81 ± 2.75	−7.05 ± 2.19	6.42 ± 1.96

## Data Availability

Data are contained within the article.
